# Exploring the Selective Vulnerability of Von Economo Neurons in Neurodegenerative Disease

**DOI:** 10.1093/geroni/igaf122.3767

**Published:** 2025-12-31

**Authors:** Maximilian Berry-Stoelzle, Yiming Liu, Georgina Aldridge

**Affiliations:** Cornell University, Ithaca, New York, United States; University of Iowa, Iowa City, Iowa, United States; University of Iowa, Iowa City, Iowa, United States

## Abstract

Alzheimer’s disease, Lewy Body dementia, and frontotemporal dementia are neurodegenerative diseases characterized by cognitive decline and neuronal loss. These diseases are diagnosed using pathological markers of protein aggregation in the brain, including beta-amyloid, tau, alpha-synuclein, and TDP-43. Studies have shown that copathologies, or the presence of more than one pathological protein occur frequently in patients, and may be a contributor to disease severity alongside synapse loss. The impact of protein copathologies on the synapses, however, remains unclear. Moreover, the selective impact of copathologies on the synapses of different classes of neurons in the human brain is not fully understood. Von Economo neurons (VENs) are a class of neurons found only in highly social animals including humans. VENs have been previously shown to be selectively vulnerable in frontotemporal dementia. We hypothesize that the presence of local copathology leads to a greater loss in dendritic spine density of Von Economo neurons. To test this hypothesis, we collected fresh tissue from the anterior cingulate during brain autopsy of consecutive neurodegenerative disease donors and control autopsies. Using Golgi-Cox staining, we visualize and quantify dendritic spine density and morphology, and neuronal complexity. Strategic dual-immunostaining of adjacent brain sections allows us to assess the impact of co-pathologies on the synapses of VENs. Our preliminary analysis has shown that the anterior cingulate is a locus of copathological build-up within both dementia and control subjects. This study provides a novel evaluation of the impact of copathologies on the synapses of vulnerable neuronal classes in neurodegenerative disease.

